# Impact of the COVID-19 pandemic on the quality of care for juvenile idiopathic arthritis patients: insights from Thailand

**DOI:** 10.1186/s13023-024-03330-7

**Published:** 2024-09-02

**Authors:** Rattakorn Pinpattanapong, Maynart Sukharomana, Sirirat Charuvanij

**Affiliations:** Division of Rheumatology, Department of Pediatrics, Faculty of Medicine Siriraj Hospital, Mahidol University, 2 Wanglang Road, Bangkoknoi, Bangkok, 10700 Thailand

**Keywords:** COVID-19, Juvenile idiopathic arthritis, Lockdown, Quality of care, Telemedicine

## Abstract

**Background:**

The COVID-19 pandemic has significantly impacted individuals with chronic conditions. This investigation assessed the quality of care provided to pediatric and adolescent patients with juvenile idiopathic arthritis (JIA) during the pandemic in Thailand.

**Methods:**

This cross-sectional analysis enrolled JIA patients aged ≤ 18 years at an academic tertiary care facility from April 2022 to March 2023. Retrospective reviews were performed, complemented by patient and caregiver questionnaires to assess the pandemic’s impact on care quality.

**Results:**

Seventy JIA patients (37 males, 33 females) with a mean age of 13.5 ± 3.1 years were included. A total of 41.4% of the caregivers reported negative impacts on JIA care due to the pandemic and the lockdown, and 31.4% of the patients experienced pandemic-related anxiety. A comparison between the pandemic and prepandemic periods revealed a higher incidence of active disease, although the difference was statistically nonsignificant (37.1% vs 14.2%, *p* = 0.106). Nonadherence significantly predicted active disease status (adjusted OR 15.04, 95% CI 2.48–91.15, *p* = 0.03). COVID-19 vaccinations were administered to 85.7% of patients; 52.8% of whom contracted mild COVID-19. Most patients (71.4%) postponed clinic visits; 36% due to lockdowns and 28% due to concerns about COVID-19 exposure in healthcare settings. The majority of patients received telephone JIA management advice from rheumatologists during the lockdown (91.4%).

**Conclusions:**

The COVID-19 pandemic and associated lockdown measures affected the care of JIA patients, impacting both physical and mental health. Nonadherence was a critical factor in disease flare-ups. Telemedicine is indispensable for patient care.

**Supplementary Information:**

The online version contains supplementary material available at 10.1186/s13023-024-03330-7.

## Introduction

Coronavirus disease 2019 (COVID-19), caused by severe acute respiratory syndrome coronavirus 2 (SARS-CoV-2), was initially identified in Wuhan, China, in November 2019 [1]. The World Health Organization declared it a global pandemic in March 2020 [2–4], which precipitated widespread lockdowns and transportation restrictions that critically impeded access to healthcare facilities [5]. Globally, hospitals responded by restricting outpatient attendance and expanding telemedicine services [6–11].

Children and adolescents with juvenile idiopathic arthritis (JIA) are vulnerable during the COVID-19 pandemic for many reasons [12]. The etiology of JIA is multifactorial including genetic predisposition and environmental triggers such as infection leading to immune system dysfunction causing chronic inflammation in the joints. Moreover, the treatment of JIA comprises of non-steroidal anti-inflammatory drug (NSAID), corticosteroids, disease-modifying anti-rheumatic drugs (DMARDs) and biologic agents. These immunomodulators may increase the risk of infection including the COVID-19.

Ensuring high quality of care in patients with JIA is imperative to maintain optimal treatment outcomes, prevent articular and extra-articular damages and good quality of life [13]. High quality of care consists of several aspects including early diagnosis with prompt referral, regular clinic visits and the treat-to-target management strategy [13–15]. Children and adolescents with JIA have been notably impacted by the pandemic and ensuing lockdowns, facing obstacles in accessing timely healthcare and specialized medications [16–18].

Biologic agents, such as anti-tumor necrosis factor, anti-interleukin-1, and anti-interleukin-6, target cytokines involved in the inflammatory process of JIA which are indicated in patients with JIA who do not respond to the combination of NSAID, DMARDs and/or corticosteroids [19–21]. The delayed access to biologic agents in JIA patients can adversely affect disease progression and increase the risk of side effects of prolonged corticosteroids treatment. Therefore, timely initiation of biologic agents is essential to improve the quality of care in patients with JIA.

This situation led to reported increases in JIA flares during the initial phase of the pandemic from 17 to 33% as reported by Conti, et al. and from 6.3 to 16.9% according to a study by Naddei, et al. [22, 23]. Moreover, enforced limitations on physical and social activities contributed to deteriorations in mental health, sleep quality, and overall quality of life [17, 18, 24]. Additionally, a considerable incidence of COVID-19 among children with rheumatic diseases was observed [25], prompting recommendations for COVID-19 vaccination in this population [26], notwithstanding vaccine hesitancy among some patients and caregivers [27].

Considering the potential disparities in healthcare systems between developed and developing nations [28, 29], this study aimed to delineate the impact of the COVID-19 pandemic and lockdown measures on the quality of care for Thai children and adolescents with JIA. Additionally, the study sought to assess attitudes toward the pandemic within the Southeast Asian context.

## Materials and methods

This cross-sectional study was undertaken at the Pediatric Rheumatology Clinic of Siriraj Hospital, Mahidol University, in Bangkok, Thailand, the country’s largest academic tertiary care center. We enrolled patients diagnosed with JIA aged 18 years or younger between April 2022 and March 2023. The International League of Associations for Rheumatology criteria [30] were employed for the JIA classifications, and demographic and clinical data were retrospectively collected from electronic health records. Pandemic era was defined by the time beyond January 2020. Clinically inactive disease was defined according to the Wallace criteria [31].

In this study, we used a self-developed structured questionnaire including single-choice responses, multiple-choice responses and rating scales*.* This encompassed questions on COVID-19 infection and vaccination experiences, care received, and psychological impacts during the pandemic and lockdown periods. Attitudes toward the pandemic’s impact on care quality and satisfaction with rheumatology healthcare communication were gauged using a 5-point Likert scale. It was self-reported completed by patient or caregivers of patients with JIA during clinic appointments as shown in the supplementary file. The Siriraj Institutional Review Board approved the study protocol (COA Si 083/2022) in accordance with the principles of the Declarations of Helsinki. Informed consent and assent were obtained from all participating parents and patients.

All the statistical analyses were performed using IBM SPSS Statistics, Version 20 (IBM Corp, Armonk, NY, USA). The requisite sample size (*n*) was determined based on the proportion (p) of JIA flares during the pandemic, as reported by Conti et al. (48.7%) [22]. Using the formula *n* = *Z*^2^ × p × (1 − p)/*d*^2^ (where the *Z* score = 1.96, p = 0.487, and margin of error *d* = 0.12), a minimum sample size of 66 JIA patients was calculated.

Descriptive statistics are presented as counts and percentages, means ± standard deviations, or medians with interquartile ranges. The chi-square test or Fisher’s exact test, as appropriate, was utilized for categorical comparisons between groups. The Mann–Whitney U test was applied to compare nonnormally distributed continuous variables. Factors associated with active disease were first examined using univariable logistic regression, with variables exhibiting *p* < 0.2 entered into a multivariable logistic regression analysis employing the enter method. Statistical significance was defined as *p* < 0.05, with confidence intervals set at 95%.

## Results

### Demographic and clinical characteristics

In this study, we included 70 patients with JIA (37 males and 33 females). The participants had a mean age of 13.5 ± 3.1 years. Enthesitis-related arthritis was the most common JIA subtype, accounting for 32.9% of the cases, followed by systemic JIA at 31.4%. Patients had a median disease duration of 4.5 years (interquartile range 3.1–6.7). The demographic and clinical details are summarized in Table 1.

**Table 1:** Demographic and clinical characteristics of children and adolescents with juvenile idiopathic arthritis (N = 70)

Characteristics	n (%), mean ± SD, or median (IQR)
Male, n (%)	37 (52.9)
Age at study visit (y), mean ± SD	13.5** ± **3.1
JIA subtypes	
Enthesitis-related arthritis	23 (32.9)
Systemic	22 (31.4)
Oligoarthritis	9 (12.9)
Polyarticular, RF−	7 (10)
Polyarticular, RF+	5 (7.1)
Undifferentiated	4 (5.7)
Disease duration, median (IQR), years	4.5 (3.1–6.7)
Hometown	
Bangkok	24 (37.1)
Outside of Bangkok	44 (62)
JIA disease status before COVID-19 pandemic	
Active	10 (14.2)
Inactive	60 (85.7)
JIA disease status during COVID-19 pandemic	
Active	26 (37.1)
Inactive	44 (62)
COVID-19 vaccination	60 (85.7)
COVID-19 infection	37 (52.8)

COVID-19, coronavirus disease; IQR, interquartile range; JIA, juvenile idiopathic arthritis; RF, rheumatoid factor; SD, standard deviation

The proportion of patients with active JIA increased from 14.2% (10/70) before the COVID-19 pandemic to 37.1% (26/70) during the pandemic, although this increase did not reach statistical significance (*p* = 0.106). Of those with active disease during the pandemic, 20 experienced disease flare-ups, while 6 continued from the prepandemic period. Systemic JIA was the most frequently flaring subtype during the pandemic (34.6%), followed by enthesitis-related arthritis (30.7%). Notably, 53.8% of patients with active disease were nonadherent to treatment during the pandemic. Multivariable logistic regression identified nonadherence as a significant predictor of active disease, with an adjusted odds ratio of 15.04 (95% CI 2.48–91.15, *p* = 0.03; Table 2).

**Table 2:** Factors associated with active disease in children and adolescents with juvenile idiopathic arthritis during the COVID-19 pandemic

Factors	Univariable logistic regression			Multivariable logistic regression		
	Crude OR	(95% CI)	*p*	Adjusted OR	(95% CI)	*p*
Duration of disease						
< 2 years	1	–	–	1	–	–
3–5 years	0.561	(0.083–3.787)	0.553	0.553	(0.029–9.760)	0.672
> 5 years	0.203	(0.028–1.468)	0.114	0.080	(0.003–1.904)	0.119
JIA subtypes						
Systemic	1	–	–	1	–	**–**
Enthesitis-related arthritis	0.770	(0.230–2.578)	0.672	0.348	(0.051–2.381)	0.282
Oligoarthritis	0.413	(0.069–2.463)	0.332	0.146	(0.011–1.957)	0.146
Polyarticular, RF−	0.578	(0.091–3.663)	0.560	0.317	(0.021–4.892)	0.412
Polyarticular, RF+	5.778	(0.551–60.605)	0.144	2.650	(0.104–67.622)	0.555
Undifferentiated	0.481	(0.043–5.401)	0.553	0.059	(0.002–2.125)	0.122
Nonadherence	6.286	(1.812–21.800)	0.004	15.047	(2.484–91.156)	0.03*
Clinic postponement	0.63	(0.219–1.813)	0.391	–	–	–
Physical activity limitation	0.313	(0.032–3.021)	0.315	–	–	–

95% CI, 95% confidence interval; COVID-19, coronavirus disease; JIA, juvenile idiopathic arthritis; OR, odds ratio; RF, rheumatoid factor

*A *p* value < 0.05 indicates statistical significance

### COVID-19 vaccination and infection

Sixty patients (85.7%) received COVID-19 vaccinations, 95% of whom received BNT162b2 (Pfizer–BioNTech). Two patients received the BBIBP-CorV (Sinopharm) vaccine, and one received the ChAdOx1 (Oxford–AstraZeneca) vaccine. The vaccine side effects were mild, with 13 patients experiencing low-grade fever and 5 reporting headaches.

Thirty-seven patients (52.8%) contracted COVID-19, all of whom presented with mild symptoms such as fever, cough, and rhinorrhea. Thirteen of these patients were treated with favipiravir. None of the children developed severe complications, such as pneumonia, acute respiratory distress syndrome, myocarditis, or multisystem inflammatory syndrome in children.

### Quality of care of JIA during the COVID-19 pandemic

Ophthalmology examinations for uveitis surveillance were slightly less frequent during the pandemic (84.2%) than during the prepandemic period (97.1%, *p* = 0.176). In contrast, the influenza vaccination rate significantly increased to 88.5% during the pandemic, compared to 62.8% during the prepandemic period (*p* = 0.019).

Clinic visit postponements occurred for 50 patients (71.4%). The reasons included lockdown-related transportation difficulties (43%), concerns about COVID-19 exposure in the hospital (41%), stable disease status (38%), and family COVID-19 infections during quarantine periods (14%).

Although a telemedicine system was available during the pandemic, only 3 patients used this platform. Instead, 64 patients (91.4%) preferred to seek medical advice via telephone from the rheumatology healthcare team.

### Psychological perception and attitude toward pandemic-related quality of care

**Fig. 1:**
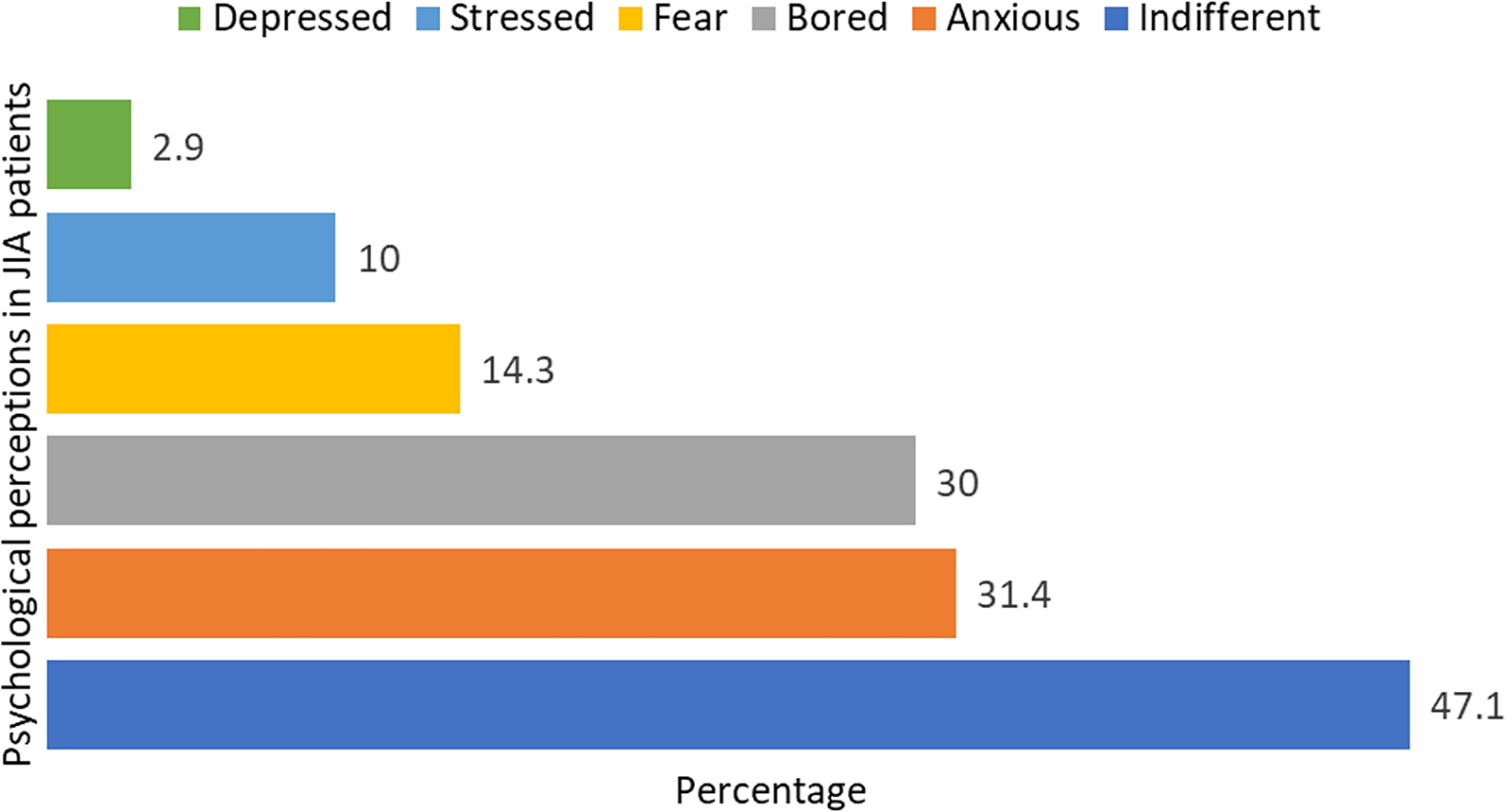
Psychological perceptions during the COVID-19 pandemic and lockdown in children and adolescents with juvenile idiopathic arthritis

Twenty-two patients (31.4%) reported feeling anxious due to the pandemic (Fig. 1). Additionally, 29 caregivers (41.4%) perceived the pandemic and lockdown as having a negative impact on the quality of JIA care. Despite these challenges, a substantial majority of caregivers (92.6%) expressed satisfaction with the healthcare services provided by the rheumatology team during the pandemic.

## Discussion

Our study underscores that the COVID-19 pandemic and associated lockdown measures substantially impacted the quality of care for JIA patients, affecting both their physical and mental well-being. A critical insight is the identification of nonadherence as a pivotal factor in JIA flare-ups, highlighting the essential role of continuous communication between patients/caregivers and rheumatology healthcare providers during such crises.

Typical JIA management encompasses both pharmacological interventions and nonpharmacological strategies such as physical therapy and regular exercise [19–21]. Naddei et al. observed an increased incidence of JIA flares during lockdowns (16.9%) compared to the preceding year (6.3%) [23]. Conversely, Miserocchi et al. reported no rise in uveitis flares among JIA patients, with no patient advised to cease immunosuppressive medications [32]. Our analysis revealed a higher occurrence of active JIA cases and flares during the pandemic, but without statistical significance.

Our findings implicate nonadherence as the predominant cause of JIA flares in our cohort, potentially exacerbated by disruptions to clinic schedules and transportation constraints during the pandemic. This result concurs with that of Conti et al., who linked JIA flares to nonadherence and extended intervals between clinic visits [22]. Another study also identified medication shortages and difficulties accessing healthcare services as contributing to nonadherence among patients requiring rheumatic and musculoskeletal disease management [5]. Notably, systemic JIA emerged as the subtype most prone to flares in our investigation. This finding is likely due to the necessity for consistent corticosteroids or biologic therapy to manage systemic inflammation, with lapses in treatment adherence leading to disease exacerbations. Horton et al. observed a notable reduction in both clinic attendance and glucocorticoid usage among JIA patients in a cohort in the United States during the first year of the pandemic [33]. Another factor contributing to disease flare-ups could be that the accessibility of tocilizumab was notably constrained during the pandemic’s first year. Tocilizumab, an intravenous humanized monoclonal antibody that targets interleukin-6 and is needed by patients with refractory systemic JIA for infusions every second week, was diverted for treating adults with severe COVID-19 pneumonia [34].

The interaction between COVID-19 infection and its impact on JIA patients warrants attention. Approximately 52.8% of our JIA cohort contracted COVID-19, manifesting primarily with mild symptoms. These findings are consistent with those of Lerkvaleekul et al., who reported similar rates of infection and severity among JIA patients [25]. Boyarchuk et al. found systemic JIA patients to be more susceptible to COVID-19, with an increased risk (odds ratio 6.16, *p* = 0.028) [35]. Despite ongoing DMARD therapy, the course of COVID-19 in our JIA cohort largely paralleled the general pediatric population’s experience with the virus [35]. None of our JIA patients developed severe COVID-19 complications, such as pneumonia, myocarditis, or multisystem inflammatory syndrome in children. With 85.7% of our cohort receiving COVID-19 vaccinations, this high uptake rate may have contributed to the predominantly mild COVID-19 symptoms observed in our study. Notably, most vaccinated JIA patients received the mRNA COVID-19 vaccine, which, according to a systematic review and meta-analysis by Hamad Saied et al. [36], is significantly associated with a reduced incidence of multisystem inflammatory syndrome in children. Furthermore, the increased rate of annual influenza vaccination observed during the pandemic suggests a growing recognition of the importance of immunization for preventing severe respiratory illnesses, a trend likely spurred by the pandemic context.

The psychological impact during and after the COVID-19 pandemic is noteworthy, with anxiety reported by 31.4% of our JIA patients. Pandemic conditions and associated lockdowns have previously been reported to lead to considerable psychological distress among patients and increased stress among parents, largely due to disruptions in daily life [37]. For example, children experienced sleep disturbances and emotional distress during lockdown periods [38], and a substantial number of JIA patients were reluctant to return to school after lockdown [39]. These findings highlight the importance of continuous postpandemic monitoring of the long-term psychological effects on JIA-affected children and adolescents.

The COVID-19 pandemic led to a rapid surge in the adoption of telemedicine across various medical specialties, including rheumatology [40, 41]. Despite its benefits, challenges such as the lack of comprehensive physical examinations and the limitations of doctor‒patient relationships have been identified [42–44]. Furthermore, the effectiveness of telemedicine in addressing psychological concerns in pediatric rheumatology patients remains a concern [45]. Although telemedicine was introduced in our hospital, its usage was limited due to internet access constraints and the difficulties experienced by caregivers in using a technology-based service. Most patients and caregivers preferred telephone communication with the health care team, valuing its simplicity and ubiquity over internet-based options. Other research has shown that caregivers with a relatively high education level, those who had missed appointments, and those facing long travel times (> 1 h) preferred telemedicine [40]. Research from a pediatric rheumatology unit in Italy, which implemented telephone consultations, demonstrates their effectiveness as a component of telemedicine services during the pandemic [32]. Another study in Canada reported high satisfaction levels with outpatient rheumatology phone visits [43]. These observations underscore the need to refine telemedicine to make it more accessible and user friendly and hence sustain its benefits beyond the pandemic [41]. Moreover, pediatric rheumatology education should be promoted for general pediatricians so that they can provide general treatment and support for JIA patients at the local hospitals of the patients' hometown in case they cannot travel to the tertiary center for regular rheumatology appointments [28, 46, 47].

This study has several limitations, particularly, its limited number of participants and single-center design. The risk of recall bias also exists, underscoring the need for a cautious interpretation of the findings. Despite these limitations, our research offers valuable insights into the real-life impact of the COVID-19 pandemic and lockdown measures on healthcare quality and system adaptability within a resource-constrained environment.

## Conclusion

In summary, the pandemic has markedly affected the quality of healthcare provided to our JIA patients, impacting both their physical health and mental well-being. Nonadherence was identified as a critical factor exacerbating disease flare-ups. The findings underscore the importance of maintaining effective communication between patients/caregivers and the rheumatology healthcare team. Looking ahead, there is a pressing need to improve telemedicine capabilities, particularly in resource-limited settings, to ensure continuous and effective care for JIA patients in the face of such global health challenges.

### Supplementary Information

Additional file 1

## Data Availability

The data that support the findings of this study are available from the corresponding author upon reasonable request. Due to privacy concerns and to protect the confidentiality of study participants, the data cannot be openly shared.

## Abbreviations

COVID-19: Coronavirus disease 2019
DMARD: Disease-modifying anti-rheumatic drug
JIA: Juvenile idiopathic arthritis
NSAID: Nonsteroidal anti-inflammatory drug
SARS-CoV-2: Severe acute respiratory syndrome coronavirus 2
