# Supplementary material for: Impact of the COVID-19 pandemic on the quality of care for juvenile idiopathic arthritis patients: insights from Thailand
Source: Orphanet J Rare Dis. 2024 Sep 2;19:320. doi: 10.1186/s13023-024-03330-7 (PMC11367977; doi:10.1186/s13023-024-03330-7)
Supplement: Supplementary file 1 — Additional file 1 [file 13023_2024_3330_MOESM1_ESM.pdf]

(For parents and patients)

No. ………………………………

Questionnaire
Impact and factors associated with juvenile idiopathic arthritis flare during COVID-19 pandemic

Part 1 Demographic data

1. Sex ❑ Male ❑ Female
2. Domicile Province…………………………

Part 2 JIA Management

1. Eye examination

1.1 Before 2020	❑ Not done	❑ Done	❑ Not sure
Result	❑ Normal	❑ Abnormal ………………….	❑ Unknown
1.2 2020-2021	❑ Not done	❑ Done	❑ Not sure
Result	❑ Normal	❑ Abnormal …………………	❑ Unknown

2. Rehabilitation

❑ No

❑ Yes ❑ Hospital……………….. ❑ Home program

3. Influenza vaccine

2019	❑ Not done	❑ Done	❑ Not sure
2020-2021	❑ Not done	❑ Done	❑ Not sure

Part 3 Communication with rheumatology healthcare team

1. Contact ❑ Never ❑ Ever
2. Contact methods ❑ By phone ❑ E-mail ❑ Others................

3. Satisfaction in contacting the rheumatology healthcare team

❑ Very satisfied

❑ Satisfied

❑ Neutral

❑ Unsatisfied

❑ Very unsatisfied

4. Follow-up at rheumatology clinic (can choose more than 1)

❑ Regular appointment

❑ Loss to follow up

❑ Postponement ❑ Self-postponement ❑ Hospital postponement

❑ Telemedicine ❑ Never ❑ Ever………………..times

5. Reasons of clinic postponement (can choose more than 1)
❑ Stable/inactive disease
❑ Have enough medication/medical supplies
❑ Transportation difficulty
❑ Contracted COVID-19 infection (self/family members)
❑ Concern of COVID-19 exposure
❑ Others……………………………………………………………

6. Satisfaction in telemedicine
❑ Very satisfied
❑ Satisfied
❑ Neutral
❑ Unsatisfied
❑ Very unsatisfied

7. Other treatment during 2020-2021

7.1 Treatment at local hospital/clinic	❑ Never	❑ Ever
7.2 Received medication at local hospital/clinic	❑ Never	❑ Ever
7.3 Self-administered/over-the-counter medication	❑ Never	❑ Ever
7.4 Missed medication	❑ Never	❑ Ever

8. Problems while receiving treatment at local hospital/clinic (can choose more than 1)
❑ No problem
❑ Lack of laboratory investigation facilities
❑ Lack of the subcutaneous injection or biologic intravenous infusion facilities
❑ Lack of medication ❑ Methotrexate (oral) ❑ Methotrexate (subcutaneous) ❑ Others…………
❑ Other problems……………………………………………………...

<u>Part 4</u> COVID-19 pandemic

1. COVID-19 test ❑ Never ❑ Ever
2. COVID-19 test ❑ Self-test ❑ Medical personnel
3. COVID-19 test method (can choose more than 1) ❑ Self ATK ❑ Rapid Ag test ❑ RT-PCR
4. Contracted COVID-19 infection ❑ Never ❑ Ever
5. Symptoms of COVID-19 infection ❑ Asymptomatic
❑ Symptomatic (can choose more than 1)
❑ Fever ❑ Cough ❑ Congestion/runny nose ❑ Dyspnea
❑ Fatigue ❑ Myalgia ❑ Loss of smell ❑ Loss of taste
❑ Diarrhea ❑ Pneumonia ❑ Others…………………

6. Treatment
❑ Home isolation
❑ Hospitel (*term used locally in Thailand during COVID-19 pandemic, referred to a hotel modified for quarantine of non-severe cases who received medical care by medial personnels comparable to a hospital)
❑ Field hospital
❑ Hospital

7. Favipiravir ❑ Received ❑ Not received

8. Modification of immunosuppressive drugs during COVID19-infection/quarantine
❑ No ❑ Yes

9. Modified immunosuppressive drugs
❑ Prednisolone ❑ Methotrexate ❑ Others………………………………….

10. Physician who modified immunosuppressive drugs during having COVID-19 infection
❑ Pediatric rheumatologist ❑ Physician taking care of patient during COVID-19 infection
❑ Parents/patient ❑ Others…………………………..

11. Experience of COVID-19 infection in family members
❑ Yes ❑ No

12. COVID-19 vaccination
❑ Not received
Reason ❑ Age <12-year-old ❑ Not interested ❑ Concern of side effects ❑ On waiting-list
❑ Received from ❑ Siriraj Hospital ❑ Others………………………………….
Dose 1 ❑ Pfizer® ❑ Sinovac® ❑ Sinopharm®
Dose 2 ❑ Pfizer® ❑ Sinovac® ❑ Sinopharm®
Vaccine side effects ❑ No ❑ Yes (can choose more than 1)
❑ Fever ❑ Headache ❑ Nausea ❑ Vomiting
❑ Fatigue ❑ Pain/ swelling at the injected site ❑ Diarrhea
❑ Skin rash ❑ Myalgia ❑ Joint pain ❑ Chest pain

13. Patient's habits during COVID-19 pandemic (can choose more than 1)
❑ Wear a face mask outdoors regularly
❑ Stay home, avoid going out
❑ Avoid using public transportation
❑ Maintain social distancing and avoid crowded area when going out
❑ Clean hands regularly with soap or alcohol gel
❑ Do not share personal items with others
❑ Eat well-cooked food
❑ Others…………………………………………………….

14. Possible causes of disease flared (can choose more than 1)
❑ Not taking medications ❑ Clinic postponement ❑ Missed clinic appointment
❑ Limited outdoor activities ❑ Prolonged sitting during studying online ❑ No exercise
❑ Others………………………………………………….

15. Attitudes toward COVID-19 pandemic (can choose more than 1)
❑ No effect ❑ Neutral ❑ Concern ❑ Bored
❑ Sad ❑ Depressed ❑ Fear ❑ Others.............................

16. COVID-19 pandemic had an effect on disease flare or worsening disease
❑ Strongly agree
❑ Agree
❑ Neutral
❑ Disagree
❑ Strongly disagree

17. Clinic postponement or missing the clinic appointment during COVID-19 pandemic had an effect on disease flare or worsening disease
❑ Strongly agree
❑ Agree
❑ Neutral
❑ Disagree
❑ Strongly disagree

18. COVID-19 pandemic and lockdown regulation made the caring of JIA patients more difficult
❑ Strongly agree
❑ Agree
❑ Neutral
❑ Disagree
❑ Strongly disagree

(สำหรับผู้ป่วยและผู้ปกครอง)

ลำดับที่....................................

แบบสอบถามโครงการวิจัย

ผลกระทบและปัจจัยที่มีผลต่อการกำเริบของโรคข้ออักเสบไม่ทราบสาเหตุในเด็ก

ในช่วงที่มีการระบาดของไวรัสโคโรนา 2019

<u>ส่วนที่ 1</u> ข้อมูลพื้นฐาน

1. เพศ ☐ ชาย ☐ หญิง

2. ภูมิลำเนา จังหวัด..............................

<u>ส่วนที่ 2</u> การรักษา

1 ตรวจตา

1.1 ก่อน พ.ศ. 2563	☐ ไม่ได้ตรวจ	☐ ตรวจ	☐ ไม่แน่ใจ
ผล	☐ ปกติ	☐ ผิดปกติ.....................	☐ ไม่ทราบ
1.2 พ.ศ. 2563-2564	☐ ไม่ได้ตรวจ	☐ ตรวจ	☐ ไม่แน่ใจ
ผล	☐ ปกติ	☐ ผิดปกติ.....................	☐ ไม่ทราบ

2. กายภาพบำบัด

☐ ไม่มี

☐ มี ☐ โรงพยาบาล.................... ☐ โปรแกรมกายภาพบำบัดที่บ้าน

3. วัคซีนไข้หวัดใหญ่

พ.ศ. 2562	☐ ไม่ได้ฉีด	☐ ฉีด	☐ ไม่แน่ใจ
พ.ศ. 2563-2564	☐ ไม่ได้ฉีด	☐ ฉีด	☐ ไม่แน่ใจ

<u>ส่วนที่ 3</u> การติดต่อหน่วยโรคข้อและรูมาติสซั่มและการตรวจติดตามที่คลินิกโรคข้อฯ

1. การติดต่อหน่วยโรคข้อฯ ☐ ไม่เคย ☐ ☐ เคย

2. วิธีติดต่อ ☐ โทรศัพท์ ☐ ☐ อีเมล์ ☐ ☐ อื่น ๆ.................

รับรองโดยคณะกรรมการจริยธรรมการวิจัยในคน
คณะแพทยศาสตร์ศิริราชพยาบาล
รหัสโครงการ 1061/2564 (IRB2)
COA no. Si 083/2022
วันที่รับรอง 26 ม.ค. 2565

(สำหรับผู้ป่วยและผู้ปกครอง)

3. กรณีเคยติดต่อคลินิกโรคข้อฯ ได้รับความสะดวกในการติดต่อ (เลือก 1 คำตอบ)

☐ ☐ ได้รับความสะดวกมากที่สุด

☐ ☐ ได้รับความสะดวกมาก

☐ ☐ ได้รับความสะดวกปานกลาง

☐ ☐ ได้รับความสะดวกน้อย

☐ ☐ ไม่ได้รับความสะดวก

4. การตรวจติดตามที่คลินิกโรคข้อฯ (ตอบได้มากกว่า 1 ข้อ)

☐ มาตามนัดทุกครั้ง

☐ ขาดการติดตาม

☐ เลื่อนนัด ☐ เลื่อนเอง ☐ ได้รับการเลื่อน

☐ ตรวจทางไกล (เทเลเมดิซีน) ☐ ไม่เคย ☐ ☐ เคย...................ครั้ง

5. เหตุผลที่เลื่อนนัดหรือขาดนัด (ตอบได้มากกว่า 1 ข้อ)

☐ ☐ อาการคงที่

☐ ☐ มียาเหลือมาก

☐ ☐ ไม่สะดวกเดินทาง

☐ ☐ มีคนในครอบครัวติดเชื้อโควิด 19/ ติดเชื้อโควิด 19

☐ ☐ กังวลสถานการณ์การระบาด

☐ ☐ อื่นๆ...

6. กรณีตรวจทางไกล (เทเลเมดิซีน) (เลือกตอบ 1 ข้อ)

☐ ได้รับความสะดวกมากที่สุด

☐ ได้รับความสะดวกมาก

☐ ได้รับความสะดวกปานกลาง

☐ ได้รับความสะดวกน้อย

☐ ไม่ได้รับความสะดวก

รับรองโดยคณะกรรมการจริยธรรมการวิจัยในคน
คณะแพทยศาสตร์ศิริราชพยาบาล
รหัสโครงการ 1061/2564 (IRB2)
COA no. Si 083/2022
วันที่รับรอง 26 ม.ค. 2565

(สำหรับผู้ป่วยและผู้ปกครอง)

7. การรักษาที่อื่นในช่วง พ.ศ. 2563-2564

7.1 รับการรักษาใกล้บ้าน	☐ ไม่เคย	☐☐ เคย
7.2 รับยาใกล้บ้าน	☐☐ ไม่เคย	☐☐ เคย
7.3 ซื้อยาเอง	☐☐ ไม่เคย	☐☐ เคย
7.4 ขาดยา	☐☐ ไม่เคย	☐☐ เคย

8. ปัญหาที่พบเมื่อรักษาหรือรับยาใกล้บ้าน (ตอบกรณีเคยรับการรักษาหรือรับยาใกล้บ้าน, ตอบได้มากกว่า 1 ข้อ)

☐ ☐ ไม่มีปัญหา

☐ ☐ ไม่สามารถเจาะแลปบางตัวได้

☐ ☐ ไม่สามารถไปฉีดยาได้

☐ ☐ ไม่มียาบางชนิด โปรดระบุ ☐ เมโธเทรกเซทชนิดกิน ☐☐ เมโธเทรกเซทชนิดฉีด ☐ ยาอื่น..............................

☐ ☐ ปัญหาอื่น ๆ
ได้แก่...

รับรองโดยคณะกรรมการจริยธรรมการวิจัยในคน
คณะแพทยศาสตร์ศิริราชพยาบาล
รหัสโครงการ 1061/2564 (IRB2)
COA no. Si 083/2022-
วันที่รับรอง 26 ม.ค. 2565

(สำหรับผู้ป่วยและผู้ปกครอง)

<u>ส่วนที่ 4</u> สถานการณ์การระบาดของไวรัสโคโรนา 2019

1. ผู้ป่วยเคยได้รับการตรวจโควิด19 ☐ ไม่เคย ☐ เคย
2. กรณีผู้ป่วยเคยตรวจโควิด 19 ☐ ตรวจเอง ☐ สถานพยาบาล/ บริการทางการแพทย์
3. วิธีการตรวจโควิด 19 (ตอบได้มากกว่า 1 ข้อ)

☐ ชุดทดสอบด้วยตนเอง (ATK)

☐ แอนติเจนเทสต์ (rapid Ag test)

☐ อาร์ที-พีซีอาร์ (RT-PCR)

4. ผู้ป่วยเคยติดเชื้อ โควิด 19 ☐ ไม่เคย ☐ เคย
5. กรณีผู้ป่วยเคยติดเชื้อโควิด 19 อาการที่มีได้แก่

☐ ไม่มีอาการ

☐ มีอาการ ได้แก่ (ตอบได้มากกว่า 1 ข้อ)

☐ ไข้ ☐ ไอ ☐ น้ำมูก ☐ หอบ

☐ ปวดเมื่อยตัว ☐ จมูกไม่ได้กลิ่น ☐ ลิ้นไม่รับรส ☐ ถ่ายเหลว

☐ ปอดติดเชื้อ ☐ อื่น..

6. การรักษาเมื่อผู้ป่วยติดเชื้อโควิด 19

☐ ดูแลตัวเองที่บ้าน ☐ สถานพยาบาลเฉพาะกิจ (ฮอสปิเทล) ☐ โรงพยาบาลสนาม ☐ โรงพยาบาล

7. ผู้ป่วยได้รับการรักษาด้วยยาฟาวิพิราเวียร์ ☐ ไม่ได้รับ ☐ ได้รับ

8. ผู้ป่วยได้รับการปรับหรือหยุดยารักษาโรคข้อฯเมื่อติดเชื้อ/ กักตัว ☐ ไม่ได้ปรับ ☐ ปรับ

9. ยาที่ผู้ป่วยได้รับการปรับหรือหยุด

☐ เพรดนิโซโลน ☐ เมโธเทรกเซต ☐ อื่น ๆ..

10. ผู้ปรับยารักษาโรคข้อฯเมื่อติดเชื้อ/ กักตัวให้ผู้ป่วย

☐ แพทย์หน่วยโรคข้อฯ ☐ แพทย์ผู้รักษาโควิด 19 ☐ ผู้ป่วย/ ผู้ปกครอง ☐ บุคคลอื่น ระบุ.................................

11. มีบุคคลในครอบครัวเคยติดเชื้อโควิด 19

รับรองโดยคณะกรรมการจริยธรรมการวิจัยในคน
คณะแพทยศาสตร์ศิริราชพยาบาล
รหัสโครงการ 1061/2564 (IRB2)
COA no. Si 083/2022
วันที่รับรอง 26 ม.ค. 2565

(สำหรับผู้ป่วยและผู้ปกครอง)

☐☐ มี ☐☐ ไม่มี

12. ผู้ป่วยได้รับวัคซีนป้องกันโควิด 19

☐☐ ยังไม่ได้รับ

เนื่องจาก ☐ อายุ < 12 ปี ☐ ไม่สนใจ ☐ กังวลผลข้างเคียง ☐☐ อยู่ในระหว่างรอ

☐ ได้รับ ☐☐ รพ.ศิริราช ☐☐ รพ.อื่น......................................

เข็ม 1 วัคซีน ☐☐ ไฟเซอร์ (Pfizer) ☐☐ ซิโนแวค (Sinovac) ☐☐ ซิโนฟาร์ม (Sinopharm)

เข็ม 2 วัคซีน☐ ☐ ไฟเซอร์ (Pfizer) ☐☐ ซิโนแวค (Sinovac) ☐☐ ซิโนฟาร์ม (Sinopharm)

ผลข้างเคียงจากการฉีดวัคซีน ☐ ไม่มี

☐ มี ได้แก่ (ตอบได้มากกว่า 1 ข้อ)

☐ไข้ ☐ปวดศีรษะ ☐คลื่นไส้ ☐อาเจียน ☐อ่อนเพลีย ไม่มีแรง

☐ปวด บวม แดงบริเวณที่ฉีด ☐ท้องเสีย ☐ผื่นแดงเล็กน้อย ☐ปวดกล้ามเนื้อ

☐ ปวดข้อ ☐เจ็บหน้าอก

13. การปฏิบัติตัวของผู้ป่วยตามมาตรการป้องกันโควิด 19 (ตอบได้มากกว่า 1 ข้อ)

☐ สวมหน้ากากอนามัยหรือหน้ากากผ้าตลอดเวลา เมื่ออยู่นอกบ้าน

☐ เลี่ยงการออกนอกบ้าน เว้นแต่จำเป็น

☐ ใช้รถสาธารณะเมื่อจำเป็นเท่านั้น

☐ เว้นระยะห่างจากคนอื่นอย่างน้อย 1-2 เมตรเมื่อออกนอกบ้าน หลีกเลี่ยงการเข้าไปในพื้นที่ที่มีคนหนาแน่น แออัด หรือพื้นที่ปิด

☐ ล้างมือบ่อยๆ ด้วยสบู่หรือเจลแอลกอฮอล์

☐ แยกของใช้ส่วนตัว ไม่ใช้ของร่วมกับผู้อื่น

☐ รับประทานอาหารที่ร้อนหรือปรุงสุกใหม่ ๆ

☐ อื่นๆ...

14. กรณีผู้ป่วยมีการกำเริบของโรคข้ออักเสบฯ คิดว่าสาเหตุของการกำเริบของโรค ได้แก่ (ตอบได้มากกว่า 1 ข้อ)

☐☐ การขาดยา/ กินยาไม่สม่ำเสมอ ☐ การมีนัดตรวจติดตามที่ห่างขึ้น ☐ การไม่สามารถมาตามนัดได้

☐ การจำกัดกิจกรรมนอกบ้าน ☐☐ การนั่งเรียนออนไลน์นาน ๆ ☐☐ การไม่ได้ออกกำลังกาย

☐☐ อื่น ๆ...

รับรองโดยคณะกรรมการจริยธรรมการวิจัยในคน
คณะแพทยศาสตร์ศิริราชพยาบาล
รหัสโครงการ 1061/2564 (IRB2)
COA no. Si 083/2022
วันที่รับรอง 26 ม.ค. 2565

(สำหรับผู้ป่วยและผู้ปกครอง)

15. ความรู้สึกของผู้ป่วยต่อสถานการณ์การระบาดฯ (สามารถตอบได้มากกว่า 1 ข้อ)

❑ ไม่มีผลกระทบ ❑ เฉย ๆ ❑ กังวล ❑ เบื่อ

❑ เศร้า ❑ เครียด ❑ กลัว ❑ อื่นๆ.............................

16. การระบาดของโควิด 19 มีผลต่อการกำเริบของโรคข้ออักเสบฯ หรืออาการที่แย่ลงของผู้ป่วย

❑ เห็นด้วยอย่างยิ่ง

❑ เห็นด้วย

❑ เฉยๆ

❑ ไม่เห็นด้วย

❑ ไม่เห็นด้วยอย่างยิ่ง

17. การนัดตรวจติดตามที่ห่างขึ้นหรือไม่สามารถมาตามนัดได้เนื่องจากการระบาดของโควิด 19 มีผลต่อการกำเริบหรืออาการที่แย่ลงของโรคข้ออักเสบฯ

❑ เห็นด้วยอย่างยิ่ง

❑ เห็นด้วย

❑ เฉยๆ

❑ ไม่เห็นด้วย

❑ ไม่เห็นด้วยอย่างยิ่ง

18. การระบาดของโควิด 19 และมาตรการล็อกดาวน์ในช่วงการระบาด ทำให้การดูแลผู้ป่วยโรคข้ออักเสบฯ ลำบากมากขึ้น

❑ เห็นด้วยอย่างยิ่ง

❑ เห็นด้วย

❑ เฉยๆ

❑ ไม่เห็นด้วย

❑ ไม่เห็นด้วยอย่างยิ่ง

รับรองโดยคณะกรรมการจริยธรรมการวิจัยในคน
คณะแพทยศาสตร์ศิริราชพยาบาล
รหัสโครงการ 1061/2564 (IRB2)
COA no. Si 083/2022
วันที่รับรอง 26 ม.ค. 2565
